# The Story of the Insane from Year to Year

**Published:** 1896-08-22

**Authors:** 


### THE STORY OF THE INSANE FROM YEAR TO YEAR.

Ninth Series.

### NORTHAMPTON COUNTY ASYLUM.

Here the numbers increased from 836 to 859 during the year 1895. There were 243 first admissions, 24 readmissions, 68 recoveries, 16 discharged relieved, and 77 deaths. The recoveries calculated in the usual way stand at 31·6, and the deaths at 9·3. Cerebral and spinal diseases account for 43 of the deaths, and consumption for 5. Old age figures as the cause of insanity in 19 of the admissions; hereditary influence in 68, epilepsy in 26, and intemperance in 21. The asylum takes in patients from the boroughs of Peterborough and Northampton; but it would seem that these boroughs did not contribute to the building of the asylum, for we find it stated that the asylum will be full of Northamptonshire cases, and that this fact should be borne in mind by the borough authorities. This evidently foreshadows a new asylum for Northampton borough, and, doubtless, this would be a better course than adding to an asylum already containing nearly 900 patients. Mr. Greene says the insane of the Northampton borough has more than doubled during the last eighteen years, and that there are now 152 patients chargeable to the borough. "No coroner's inquest was held during the year, and no serious accident of any kind took place." The cost per head per week is 7s. 9½d.

### CHESHIRE COUNTY ASYLUM, PARKSIDE.

At the beginning of the year there were 718 patients in this asylum, and at the close the number had fallen to 703. There were 129 first admissions, 16 readmissions, 51 recoveries, 16 discharged relieved, and 71 deaths. Calculated on the admissions the recoveries stand at 36·9, and, calculated on the average number resident, the deaths stand at 9·8 per cent. Thirty of the deaths were caused by cerebral and spinal diseases, 15 by consumpton, 6 by typhoid fever, and 1 by enteritis. Intemperance in drink accounts for 27 of the year's admissions, hereditary for 28, epilepsy 11, and domestic trouble for 17. There was an outbreak of typhoid fever, affecting 25 cases, and, as already stated, six of them died. It is curious to notice how almost invariably the mortality from these febrile diseases is above the average among the insane. The outbreak was traced to the pollution of drinking water by a faulty sewer. A new infectious hospital was opened during the year. Dr. Sheldon and the assistant medical officers, Drs. Cooke and Laing, continue their courses of instruction to the staff, and a high proportion of the nursing staff has passed the nursing examination of the St. John's Association. We also learn that a pension scheme is under consideration by the committee, and it is most earnestly to be hoped that the scheme will be carried to a successful issue. For the subordinate staff the highest pension permissible by the Lunacy Laws provides a certainty for old age, but is scarcely sufficient to rely on entirely, and we venture to urge the staff to join the National Pension Fund. The two pensions combined are sufficient. The committee record their continued satisfaction with the management of the asylum by Dr. Sheldon. The weekly cost per head is a fraction under 9s.

### LEICESTER BOROUGH ASYLUM.

An increase from 529 to 561 has to be chronicled at this asylum. There were 115 first admissions and 23 readmissions; 48 were discharged recovered; 12 discharged relieved, and 32 died. The recoveries stand at 38 per cent. of the admissions, and the deaths at 5 9 per cent. on the average resident. Of the deaths 14 were caused by cerebral and spinal diseases, and 5 by consumption. Drink accounts for 15 of the year's admissions, hereditary influence for 31, and domestic trouble for 6. Dr. Finch had under treatment during 1895 the large number of 112 epileptics, and 10,376 fits were registered. The question of enlarging the asylum has been spoken of, and in the meantime all the out-borough patients have to be removed. Dr. Finch calculates that in ten years' time there will be 700 patients to provide for, and his estimate is more likely to be under than over the truth. The Committee record "their appreciation of the highly satisfactory manner in which Dr. Finch and the officers have discharged their respective duties." The weekly cost of maintenance is a fraction over 10s. per head.

### SURREY COUNTY ASYLUM AT BROOKWOOD.

On January 1st this asylum contained 1,074 patients, and at the end of the year the number had risen to 1,099. There were 249 first admissions, 37 readmissions, 84 recoveries, 24 discharged relieved, and 139 deaths. The recoveries stand at 31·34 per cent. on the admissions, and the deaths at 12·69 per cent. on the average resident. Fifty-four of the deaths were from cerebral and spinal diseases, 22 from consumption, 11 from pneumonia, and 8 from enteritis. Turning to Table X., which shows the causes of insanity in the year's admissions, we find intemperance in drink credited with being the cause in 50 cases, hereditary influence in 92, domestic trouble in 8, and religion in 5. Dr. Barton comments on the unfavourable nature of the cases admitted, and he thinks that only 96 out of the 286 can be regarded as curable. Such statements by a careful medical superintendent like Dr. Barton, show what utter nonsense is talked by the advocates of acute hospitals, and how little confidence can be placed in the predictions of those who advocate these innovations. The female side of the asylum was overcrowded during the year, and this must have greatly added to the trouble and worry of the superintendent's life. There are 17 idiot children in the asylum, and Dr. Barton does not think the asylum a suitable place for them. This is self-evident, and a sub-committee has been appointed to consider the matter. Why not build a separate block for these children, as has been done at Northampton and Hampshire with much success? The weekly charge to the unions is 10s. per head.

